# Philadelphia Obstetrical Society

**Published:** 1889-04

**Authors:** J. M. Baldy

**Affiliations:** Secretary


### Society Reports.

### *PHILADELPHIA OBSTETRICAL SOCIETY.*

Reported by J. M. Baldy, Secretary.

THURSDAY, FEBRUARY 7, 1889.

Dr. Theophilus Parvin in the chair.

Dr. Hoffman: *A Case of Pyosalpinx:*

I shall go into the history of this case with rather more attention to detail than would be warranted, were it not for some features of previous history and treatment which render it in some respects more than ordinarily instructive.

Mrs. G., aet. 28, came to me for examination in August, 1888. Her history at the time was unsatisfactory, giving simply record of a discharge for a long while and great pelvic pain, especially on the right side. A miscarriage was admitted. Examination showed a painful mass, bound down to the right cornu uteri, so tender that exact mapping out was impossible. I doubt, however, whether even under an anesthetic an accurate diagnosis could have been made of the condition as revealed by operation, owing to the involvement of intestine. Intestinal adhesions always increase the size of ovarian and tubal tumors, rendering the decision of absolute extent impossible. The left side also was evidently involved, but was not so painful as the right. I made no great effort to determine the extent or nature of this involvement, inasmuch as her condition was evidently one for operation, as I told her, saying at the same time that other treatment would be entirely useless. She left my office crying, and I saw no more of her until two days before operation, December 18, 1888. I then ascertained that after seeing me she had gone to four other physicians, one whose diagnosis was uterine displacement, and whose treatment was the introduction of a pessary, strangely enough with the result of apparent relief. She then visited the clinic of the

Woman's Hospital, and was comforted with the assurance that there was nothing the matter with her, and that she should go home and have a baby. She received like advice at the University. An up-town specialist, into whose hospital she afterward went, with the intention of operation "for a ruptured blood-vessel," the then diagnosis, told her there was no necessity for operation, advising her that she was wise not to have submitted to it at my hands, promising her at the same time that she should have a baby, and to promote this end, the uterus was curetted. Time wearing on, she again visited her last adviser, who now made the diagnosis of a ruptured blood-vessel, and advised operation for its "tying." At this juncture, she again fell into my hands, much reduced from excessive hemorrhage for three weeks. I did not examine her, but urged immediate operation, to which she consented. Two days later, abdominal section was done. Assisted by Dr. J. Price, the right side was first explored, and the ovary and tube found everywhere densely adherent. Enucleation was accomplished with much difficulty, and when attempt was made to apply the ligature, the tissues were found so rotten, that the blood-vessels alone withstood the tension of the silk. The right cornu uteri was so involved, that it was simply a mass of abscess tissue, and had to be curetted in order to free it from the necrosed portion. The bleeding was now so profuse, that the application of a new ligature was necessary. The second ligature was cast wholly about uterine tissue. The left side was then examined, and found even more extensively involved than the right, but without the presence of pus. The adhesions were more dense, but the tissues not being necrosed, there was no difficulty in obtaining a good pedicle. The involvement of intestines was so great that I fully expected a fecal fistula to result, in spite of careful suturing. The right tube contained pure pus. A drainage-tube was introduced, and kept in the incision a little more than a week. The only complications were severe inflammation of the bladder, and phlebitis of left intra-saphenous vein. The patient is at present moving about her room.

The history of her trouble since obtained is interesting. She was married at sixteen; six weeks after her marriage her husband deserted her. Her baby lived seven months, going blind some time before its death, having sores, and becoming very emaciated. At this time she began to have a bad discharge, which inflamed her private parts, which were also much swollen. Had great pain on urination. She did not have intercourse for twelve months after the birth of her baby. She then contracted an alliance with another man, and afterwards, within three years, had two miscarriages, and a still-birth at eight months. Her hair has fallen out, but, aside from this, she has had no other signs of syphilis. The question here arises: What was the origin of the pelvic trouble? Was it gonorrhea, syphilis, abortion, or the after-effects of labor? That there are chances for believing, according to the various aspects of the case, that perhaps one, then the other, of all these agencies entered into the causation of the disease, will, I believe, be not disputed. Whether or not it is so conceded, matters very little, however, so far as this case is concerned. That all these factors may bring all the various forms of pelvic disease, cannot be rationally disputed. That we can dogmatically affirm that any pelvic lesion is brought about by any one of these causes, with the exception of gonorrhea, as proved by the presence of the gonococcus, is not to be for a moment entertained. The fact is, we can have the symptoms of pyosalpinx simulated in all its essentials by an entirely different condition, to-wit: tubercular disease of the appendages. To consider the relative frequency of each of these factors in the production of pelvic disease, it is not my purpose, but it requires more than the dogmatic assertion of any operator to prove that this, that, or the other cause is always at the bottom of the lesion. But while the etiology of the trouble may be obscure in well-defined lesions, like the one under observation, the diagnosis ought, in most instances, to be made; or, if it cannot, the questions concerning it should be gone over, chances for error discussed, and once for all have it confessed that the subject of pus-tubes and ovaries is not too hackneyed for consideration before a society such as this. We read of the infallible bimanual examination which is able to pick up a Fallopian tube, or distinguish varicose veins in the broad ligament, and then suddenly are confronted by failure such as I have here recorded. The fact stands out that the diagnosis insisted upon by the men who are accustomed to claim everything, is a myth, and, as Mr. Tait says, fit only for library papers. It is incredible that the diagnostician who can recognize, map out, and differentiate the Fallopian tubes, should fail to recognize a mass the size of a small fist. Let us by all means have diagnosis, but let it be *diagnosis*, not myth. In this case, the diagnosis, promises, and treatment well nigh lost the woman her life. She wanted a baby, and she was promised one. She nursed the delusion for three months. Promises may hold patients; they do not work cures, nor save reputable medicine the slur and suspicion of quackery. I believe, in the present case, that the curetting was directly responsible for the involvement of the uterine tissue. In the presence of tubal disease, there is no excuse or palliation for the use of the curette. I believe that the so-called operation of "dilating and scraping" is responsible for much tubal and ovarian trouble, that would otherwise remain quiescent. I have now a case under observation where this procedure was resorted to without relief; then electricity was tried, and to-day the patient is worse than ever, with operation her only relief.

DR. JOSEPH PRICE: *A Year's Work in a Maternity Hospital.*

In making this report, I desire briefly to call attention to the amount of work done, the routine treatment of patients, and a few alterations which have taken place in the building. During the year 1888, there were 184 deliveries in the Retreat. Of these patients, sixty-nine were primiparas. There were 186 children born, including two sets of twins; nine of these infants were still-born, 102 were males, eighty four were females. There were thirteen forceps deliveries. Labor was induced in two cases in the eighth month—in one case due to contracted pelvis, and in one to the presence of a large uterine tumor. There have been no deaths of mothers in the Retreat for a period of nearly five years, furnishing a series of 540 deliveries without a death, the last death being from puerperal convulsions in a patient suffering from chronic Bright's disease, and who had had convulsions in five previous labors. Since this death there has not been a case of puerperal septicemia in the institution. The great success attending the work of this maternity is due to the strict enforcement of the law of cleanliness. Everything and everybody in the house is clean, and jealously kept so. This system was enforced by Dr. Goodell, and has been carried out on the lines laid down by him. The routine treatment of patients is as follows: The patient, on entering the house, is given a hot soap bath, dressed in clean underclothing, and given a clean bed in the waiting ward. If necessary, a laxative is given, and the bowels kept soluble during her waiting period. Thereafter, until her confinement, she is obliged to take a least two hot soap baths per week, and to wear clean clothes. She is allowed to do such light work about the house as the physician may deem advisable, and is encouraged to take as much open-air exercise as circumstances will permit. Every effort is made by the officers and employees of the institution to make it as cheerful and homelike as possible. When ready for the delivery-room, the patient is again given a hot soap bath and an enema, and a vaginal injection of 1 to 2000 bichloride of mercury solution. She is clothed in clean night-robe and drawers, and placed upon a new, clean delivery-bed. Scrupulous cleanliness is observed in all manipulations of the patient, and after delivery a second vaginal injection is given, and a vaginal suppository of iodoform is introduced. The patient's person is carefully cleaned, all soiled clothing is removed, the binder applied, a clean set of night-clothes put on, and the patient placed in a new, clean bed in the ward. All of the soiled articles are immediately removed from the delivery-room, and a new bed made up for the next patient. The patients in the ward are carefully observed by the nurses, but no unnecessary handling or interference indulged in. The patients remain in the ward until they are able to be up, when they are removed to the convalescent ward. As the ward is emptied, the beds are burned, and all the bedding most carefully cleaned. No soiled linen (as draw-sheets, diapers, napkins, or other articles of clothing,) is allowed to remain in the ward; but, when soiled, is immediately placed in a covered receptacle and removed from the ward and building. No sponges, wash-rags, or absorbent cotton are used in the house. Corrosive jute supplies the place of these articles; being clean, soft, remarkably absorbent and cheap, it is destroyed immediately after use. The pads used to absorb the lochia are also composed of jute, and are likewise destroyed after use. The beds in the wards are of new straw. All discharges from the delivery-room are immediately burned. All bedding soiled beyond cleansing, or contaminated by purulent or specific discharges, is likewise burned. In short, every effort is made to keep the house perfectly pure and sweet. The arrangement of the house permits of rotation in the use of the wards, so that a ward once emptied is not again used until three others have been filled. In the meantime, it is most carefully and scrupulously cleaned and thrown open to the atmosphere. A similar system is pursued in the convalescent wards and delivery-room. A few alterations in the building have very markedly increased the effectiveness of the institution and the comfort of its inmates. In the first place, the bath-rooms and water-closets have been removed from the building proper, and placed in the towers in the rear. The plumbing is as near perfect as modern sanitary science can make it. The verandas have been enclosed in glass, forming large, light, airy corridors about the rear of the building, and furnishing a distinct, circulating atmosphere between the house proper and the wards and the water-closets. The ventilation of the entire building is simply perfect. The capacity of the house at present is about fifty patients per month, and, when a few contemplated changes are made, the capacity will be doubled and the institution rendered as nearly an ideal maternity hospital as is practicable.

Dr. Wm. Goodell said it had always been a matter of great regret to him that he did not adopt this system a year, or a year and a half, before he did. He supposed it was partly due to the conservatism of old age, and partly to a series of some forty deaths from bichloride poisoning he had collected. Tarnier's reports of the results following the use of this agent so impressed him that he was led to make the change. Before he adopted the system which has just been detailed by Dr. Price, he had once as many as five deaths in about 150 cases, four of these due to septicemia. Latterly, hardly a year would elapse without the occurrence of one or two deaths. When he first started, everything about the institution was new and clean, and for several years he had the best record of any maternity hospital in the world. After the building and articles had become old, deaths began to occur. He tried carbolic acid, but it proved of little value. After beginning the use of corrosive sublimate injections, iodoform suppositories and antiseptic pads, he did not have a death from septicemia. The only death was one from Bright's disease of the kidneys. During this time he had been consulted perhaps a dozen times in the course of a year, to see women dying from puerperal septicemia. He thought that in private practice it would not be needful to follow out so strictly the details of the method, as it is practised at the Preston Retreat. For instance, the antiseptic pad and the iodoform suppositories might be done away with. He believed, however, that every practitioner should syringe out the vagina, both before the birth of the child and after complete delivery, with a bichloride solution of 1 to 2000. The hands should also be disinfected. He was called in consultation by a physician in the country who had had four or five deaths from sepsis in a short time. He found he had been treating a case of phlegmonous erysipelas. He knew of another physician who had lost, he thought, seven cases, certainly five, from dressing a sloughing case of erysipelas. Antiseptic measures would probably have saved all these cases.

DR. HENRY LEAMAN would call the attention of those who have the opportunity of observing the physiological processes of labor, to one point, viz.: presentation. It is very difficult to accurately determine the presentation, particularly of the face, brow and posterior presentations. These observations should be verified by examination of the abdomen previous to labor, and the location of the fetal heart sounds. They should also be confirmed by observation of the position of the head in the act of delivery. A mistake is readily made in posterior presentations. Posterior presentations are, he thinks, more frequent than we are in the habit of considering them. His object in speaking was to say that every case of labor was a case for the minutest observation. There was another point which he thought should be observed—that was the hour of the day at which labor occurs. There is, he thought, probably some connection between arterial pressure and the time of delivery. In recording the hour, there would be an allowance to be observed in cases where the forceps were used. There was another point not mentioned, and that was the position of the succedaneum and its extent. These have to do with the natural process of labor, and aid in determining the presentation.

DR. J. PRICE said he was as anxious about a labor as he was about a section, when he read reports of maternity hospitals with a mortality of from two to twenty-seven per cent; this troubled him not a little now that he controlled a large maternity hospital, one in which Dr. Goodell had left a record of 275 cases without a death. He visits a labor case as frequently as he does a drainage after abdominal section. When this hospital was new, Dr. Goodell had a run of 250 cases without a death from any cause. This was the longest run of any institution at that time; after this, deaths began to occur; later, he adopted the gospel of cleanliness, and with what result he has just told you; the results are now precisely the same as he left them. In regard to Dr. Hirst's question as to whether the same results might not be obtained by simpler methods, Dr. Price said that they did not differ much in regard to the use of solutions, and that portion of the treatment. The toilet of the house was perhaps just as systematically carried out at the Philadelphia hospital as at other institutions. The pad which he had shown would hold a pint of fluid. It saved an immense amount of laundry work. It was now coming into use as a menstrual pad, and was very convenient for ladies traveling. In private practice the mortality was greater among the rich than the poor. Among the poor he had had 700 deliveries without a death. He thought the difference was in the water-closets which the better classes had in their houses. The mortality throughout the country was large. In a small town in Ohio, with a high elevation and beautifully located, he had recently known of two deaths from septicemia. Last Summer he had been called to see puerperal cases nine times, and all died.

